# Digital Microfluidics for Manipulation and Analysis of a Single Cell

**DOI:** 10.3390/ijms160922319

**Published:** 2015-09-15

**Authors:** Jie-Long He, An-Te Chen, Jyong-Huei Lee, Shih-Kang Fan

**Affiliations:** Department of Mechanical Engineering, National Taiwan University, Taipei 10617, Taiwan; E-Mails: D93b47202@ntu.edu.tw (J.-L.H.); andy80103@hotmail.com (A.-T.C.); b00505004@ntu.edu.tw (J.-H.L.)

**Keywords:** microfluidic chips, digital microfluidics, single cell analysis, genetic screening, assisted reproductive technologies

## Abstract

The basic structural and functional unit of a living organism is a single cell. To understand the variability and to improve the biomedical requirement of a single cell, its analysis has become a key technique in biological and biomedical research. With a physical boundary of microchannels and microstructures, single cells are efficiently captured and analyzed, whereas electric forces sort and position single cells. Various microfluidic techniques have been exploited to manipulate single cells through hydrodynamic and electric forces. Digital microfluidics (DMF), the manipulation of individual droplets holding minute reagents and cells of interest by electric forces, has received more attention recently. Because of ease of fabrication, compactness and prospective automation, DMF has become a powerful approach for biological application. We review recent developments of various microfluidic chips for analysis of a single cell and for efficient genetic screening. In addition, perspectives to develop analysis of single cells based on DMF and emerging functionality with high throughput are discussed.

## 1. Introduction

Multicellular organisms are composed of varied cells grouped into specialized tissues and organs, which typically comprise cells of diverse types present in widely varying abundance. The single cell is the basic structural and functional unit of a living organism. The most critical knowledge for biological and biomedical science is constructed from research on cell biology [1,2]; this information about cell functionality and behavior has been applied in many clinical and biomedical applications [3], such as drug development, disease diagnosis, cancer research and assisted reproductive technology (ART).

Traditional cell assays to study cell differentiation, gene expression and drug response were focused mainly on a population of cells. For an average result of multiple cells, the outcome of all cells is assumed to be homogeneous, but several authors have found cellular heterogeneity or multi-modal distributions in a cell population [4,5,6]. The heterogeneity might arise through genetic or non-genetic processes, which induce distinct cellular decision-making [7]. If an average response of multiple cells is taken to be representative of a typical population, cellular heterogeneity might result in a misleading interpretation [8]. Analysis of a single cell indicated that individual cells that form a cell population might have a complicated distribution. This information is ignored in a traditional cell assay, which emphasizes evaluating the mean of a cell population [9]. To understand the variability from cell to cell and to improve the clinical and biomedical applicability, development of new approaches to isolate, to manipulate, to treat and to analyze single cells are required. Analysis of single cells has become a key technique in biological and biomedical research, as one can thereby analyze individual cells within a cell population [1,7].

Various techniques for analysis of a single cell have been developed, such as flow cytometry and microfluidic chips [10]. Flow cytometry, which has been under development for many years, has become a mature technique for single-cell analysis. In particular, fluorescence-activated cell sorting (FACS), builds upon flow cytometry in order to sort single cells that are tagged with specific fluorescent markers [11,12]. Flow cytometry is, however, a complicated and costly system: it cannot support analysis of cells in real time in their natural environment. Furthermore, integration of an entire assay based on a single cell typically entails processes such as cell manipulation, treatment and final detection. To overcome these problems, integrating various methods of cell manipulation with microfluidic lab-on-a-chip (LOC) platforms, also known as microfluidic chips, has become a major activity in assays of single cells [8,11,13,14,15]. Microfluidic chips could provide efficient genetic screening through a well-controlled microenvironment for analysis and treatment of a single cell. On these platforms, isolation of a single cell, purification of mRNA and subsequent multiplex quantitative polymerase chain reaction (qPCR) in real time must be effected on a chip [16]. Single-cell genomics, transcriptomics, epigenomics and proteomics will allow many enduring questions in biological and biomedical sciences to be answered [17,18,19,20]. Digital microfluidics (DMF), the manipulation of individual droplets by electric forces, is one of the particularly important techniques for single cell manipulation and analysis on a microfluidic chip. In this paper, we review recent developments in DMF chips for analysis of a single cell and efficient genetic screening, which involve manipulation of a single cell, next-generation sequencing (NGS) and assisted-reproductive applications. We conclude with views on the future development of DMF chips for analysis of a single cell and discuss how this emerging efficient tool can advance biological and biomedical research.

## 2. Microfluidic Chips for Analysis of a Single Cell

In an organism, a single cell is small: its volume is about 1 pL. Cells constituting a tissue or organ are complicated and diverse. An extracellular matrix (ECM) provides structural and biochemical support of the structural connection. To analyze a single cell on a chip, a microfluidic chip typically entails integration of four functions: (1) cell sorting, through microwells [21,22,23,24,25], traps [26,27,28,29], optical tweezers [30,31,32,33] or dielectrophoresis (DEP) [34,35,36,37] to isolate a single cell; (2) cell manipulation, through a traditional syringe pump [38] or an electrowetting-on-dielectric (EWOD) technique [39]; (3) cell lysis, through a mechanical [40], chemical [24] or electrical [23,41,42] process; and (4) analysis of an individual cell, such as with PCR [21,43] or NGS [44].

Microfluidic chips have been applied in biological and biomedical research, including culturing, sorting, patterning and genetic screening of single cells for clinical diagnostics [45,46,47,48,49]. These platforms have become a promising tool for efficient genetic screening through analysis of single cells, because these microfluidic chips can provide rapid, real-time and automated analysis. Gossett *et al.* [50] demonstrated an automated microfluidic technique capable of probing single cells. A rapid assay of the deformability of native populations of leukocytes and malignant cells in pleural effusions has been enabled on this chip. Guan *et al.* [51] introduced a new microfluidic chip with real-time feedback control to evaluate single-cell deformability, which was used to discriminate different kinds of cells for cancer diagnosis [30]. Guo *et al.* [52] produced a microfluidic chip to distinguish red blood cells containing parasitic *Plasmodium falciparum* from uninfected cells. Several microfluidic chips have been generated to capture single cells and to measure the impedance of the cells, such as human cervical epithelioid carcinoma (HeLa) cells [53,54] or circulating tumor cells (CTCs) from blood [55,56]. Kurz *et al.* [57] reported a microfluidic chip to trap single cells and to measure the impedance for the monitoring of sub-toxic effects on cell membranes.

**Figure 1:**
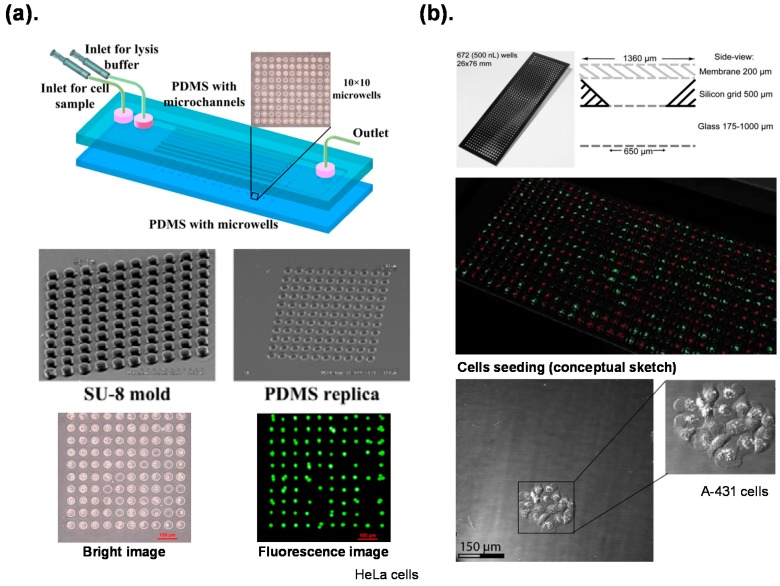
Individual cells isolated on a chip with microwells described in: (**a**) Jen *et al.*, 2012 [24]; (**b**) Lindstrom *et al.*, 2009 [21]. Reproduction of the figures has been made with permission from Multidisciplinary Digital Publishing Institute and The Royal Society of Chemistry.

The method most frequently used to isolate a single cell is physical separation. At designed physical boundaries, an individual cell is isolated, captured and sorted with mechanical structures on a chip. Capturing an individual cell with microwells is an attractive strategy, because it is simple and easily operated. Jen *et al.* [23,24] reported microfluidic chips with arrays of microwells that isolated individual cells and provided chemical and electric lysis of a single cell with high throughput (Figure 1a). Lindstrom *et al.* [21,22,58,59] developed a novel microplate with microwells for efficient analyses of single cells. This platform allowed each single cell to be cultivated and analyzed individually for reprogramming factor evaluation on stem cells [22], PCR amplification and genetic analysis [21] (Figure 1b).

**Figure 2:**
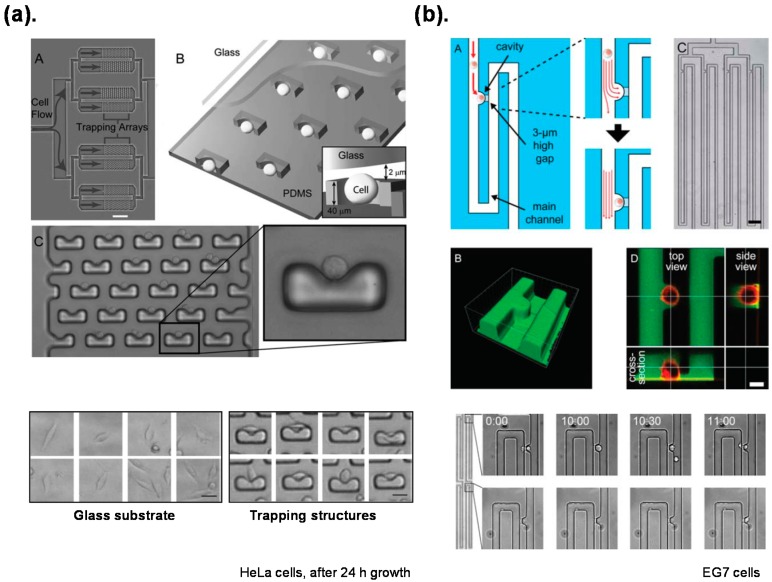
Individual cell isolated on a chip with microfluidic hydrodynamic traps described in: (**a**) Di Carlo *et al.*, 2006 [26]: (**A**) A photograph of the cell trapping device; (**B**) A diagram of the device and mechanism of trapping; (**C**) A high resolution micrograph of the trapping device; (**b**) Kobel *et al.*, 2010 [60]: (**A**) Schematic illustration of the single cell trap; (**B**) A three-dimensional reconstruction image of the cell trap; (**C**) An array of the single cell traps; (**D**) An orthogonal view of a fluorescently labeled single cell. Reproduction of the figures has been made with permission from The Royal Society of Chemistry.

Microfluidic hydrodynamic traps combine dynamic cell isolation with prospective high throughput on a chip [60,61]. Di Carlo *et al.* [26,62] produced a dynamic platform that allows culture of a single cell with a consistent environment and dynamic control of individual cells (Figure 2a). Kobel *et al.* [60] reported a microfluidic chip with efficiency of trapping a single cell enhanced up to 97% (Figure 2b).

**Figure 3:**
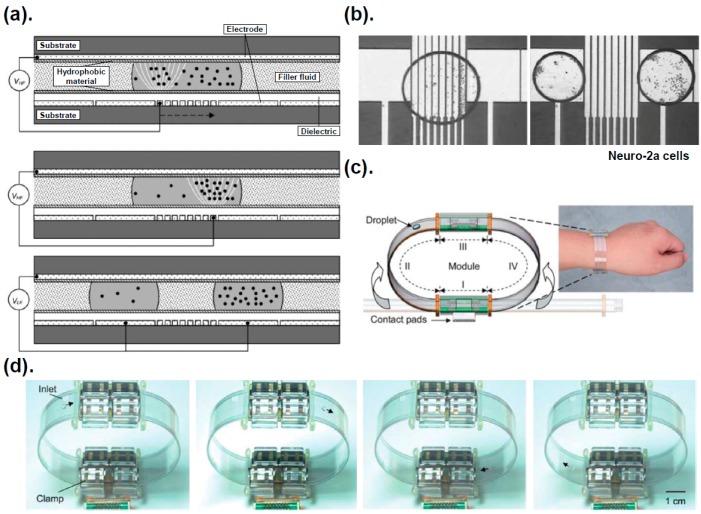
Dielectrophoresis (DEP) forces exerting on the suspended particles described by Fan *et al.*, 2008 [39]. (**a**) A parallel-plate device with square and strip electrodes to manipulate droplets and a particle; (**b**) DEP forces exerted on suspended particles including mammalian cells (Neuro-2a); (**c**,**d**) Prototype of EWOD-based, continuously microfluidic module for a portable system reported by Fan *et al.*, 2011 [70]. Reproduction of the figures has been made with permission from The Royal Society of Chemistry.

Compared with use of mechanical structures, isolating a cell with an optical or electric force is a contact-free method that eliminates deformation and damage of cells [63,64]. Here we focus more on the electric forces, including DEP and EWOD. The DEP force generated with a non-uniform electric field interacting with polarized, suspended particles [65,66,67] has been widely used to manipulate cells [39,63,68,69]. The DEP force is classified as positive DEP (pDEP) and negative DEP (nDEP) based on the polarizability of the particles or cells and the liquid. Fan *et al.* [39] used DEP forces to concentrate suspended particles in a liquid droplet with dielectric-coated electrodes patterned on a plate (Figure 3a). Creating two droplets with mammalian cells and polystyrene beads at distinct concentrations was achieved with DEP and EWOD (Figure 3b).

## 3. Digital Microfluidic Chips and Biological Application

Digital microfluidics (DMF), according to which tiny droplets are manipulated with electric forces, including EWOD and DEP, have been confirmed to be a powerful platform for reagent addition, droplet transmission, solution mixing, splitting and dispensing for biological application [70,71,72,73,74,75,76,77,78,79,80]. For a simple configuration of device and easy modular interfaces, portable and wearable DMF systems with assembled modules for continuous actuation of droplets were demonstrated [70] (Figure 3c).

A DMF chip with arrays of electrodes is typically fabricated on indium tin oxide (ITO) glass plates using photolithography and wet etching. The patterned ITO electrodes are then covered with a dielectric and a hydrophobic layer [16,39,73,81]. The advantages of DMF include ease of fabrication, simple device structure, small consumption of reagents, easy integration with analytical instruments and prospective automation. Thus, DMF has become highly suitable for biological application. Barbulovic-Nad *et al.* [80] introduced a DMF chip to implement cell-based assays; the platform was demonstrated to be advantageous for cell-based assays because of potential for automated manipulation of multiple reagents. Vergauwe *et al.* [78] reported a DMF chip for homogeneous and heterogeneous bio-assays with great analytical performance capable of medical applications. Kumar *et al.* [75] demonstrated the first use of a DMF technique for individual protoplasts from *Arabidopsis thaliana* plants. Shih *et al.* developed the first DMF chip capable of cell impedance sensing [76]; they also integrated droplet-in-channel microfluidics with DMF to develop a novel chip to perform complicated assays [81].

This work demonstrates that DMF chips would be a generic and powerful platform for the biological assays, including drug screening, immunoassays, analysis of single cells and digital PCR. This promising new technique might allow the efficient genetic screening based on a single cell to become a reality.

## 4. Digital Microfluidic Chips for Genetic Screening

Investigating gene expression and developing genetic screening at a level of a single cell provides an important capability to resolve the problem of disease etiology, cancer pathology and other biomedical applications [82]. Traditional methods of genetic screening require a large amount of sample for an analysis, which typically decreases the sensitivity and accuracy on analysis of only a single cell [83,84]. Various microfluidic techniques have been developed to address this problem. Digital polymerase chain reaction (digital PCR) platforms have measured DNA or cDNA of a single cell [85,86], but challenges persist in treating the integration of varied programs for genetic analyses of a single cell on a device, including cell sorting, manipulation, lysis, PCR and genetic screening. Within the past decade, microfluidic chips have become one of the most powerful platforms to achieve efficient genetic screening at the level of a single cell [16,87]. Toriello *et al.* [88] and Bontoux *et al.* [89] reported a polydimethylsiloxane (PDMS) device to analyze the gene expression of single cells, and Marcus *et al.* [90] developed a microfluidic chip with integrated flow that conducted cell capture, lysis, mRNA purification, cDNA synthesis and purification but with a complicated auxiliary system for control.

**Figure 4:**
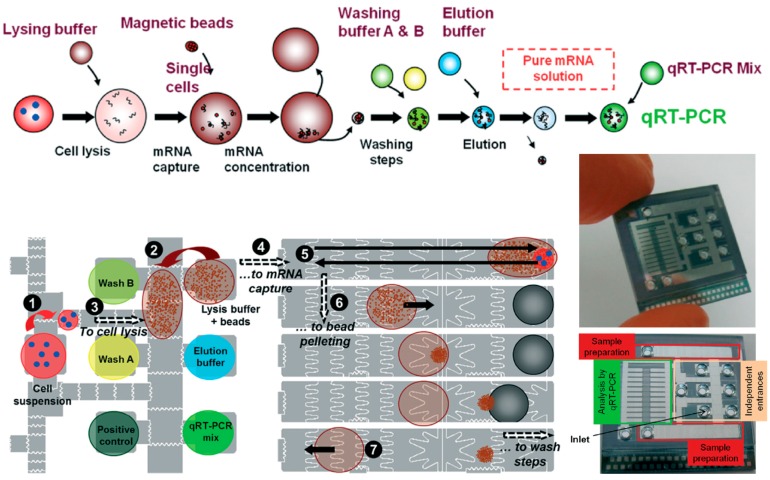
An integrated EWOD system for genetic analysis based on a single cell described by Rival *et al.*, 2014 [16]. The lysis and mRNA capture steps: (1) A droplet containing a few cells is dispensed; (2) A droplet of lysis buffer and magnetic beads is dispensed; (3) A droplet with the cells and a droplet with the beads are merged for cell lysis; (4) This merged droplet is moved to the “sample preparation” electrodes for operating magnetic beads; (5) The droplet is moved back and forth to enable bead mixing and mRNA capture; (6) The droplet is moved towards the magnet, the beads are concentrated to result in bead extraction; (7) An empty droplet is moved towards the waist. Reproduction of the figures has been made with permission from The Royal Society of Chemistry.

As mentioned above, the DMF chip has advantages of ease of fabrication, simple supporting instrumentation and prospective automation. Rival *et al.* [16] described an integrated and automated EWOD system to perform a complete workflow from the isolation of a single cell to a genetic analysis (Figure 4). DMF is becoming a powerful approach for biological applications, even enabling sample preparation for PCR to develop an efficient genetic screening platform based on a single cell [16,91,92].

## 5. Efficient Genetic Screening for Assisted Reproductive Techniques

The early development of a mammalian embryo is a complicated process involving an upheaval of a transcriptional architecture [93,94,95]. In applications, human *in vitro* fertilization (IVF) is an important scientific achievement in the twentieth century, but until recently there has been little knowledge of regulatory mechanisms in genes of early mammalian embryos. The early embryo undergoes cleavage divisions in a series from two cell, four cells, eight cells, morula, even to blastocyst. A platform for genetic screening based on a single cell could provide critical knowledge to clarify regulatory mechanisms of genes in early mammalian embryos [93]. This emerging efficient technique would benefit a biomedical approach, such as assisted reproductive technology (ART).

The microfluidic ART platforms under development are focused on simulation of the Fallopian tube to optimize the IVF, especially the early embryo culture *in vitro* [96,97,98]. Pre-implantation genetic diagnosis (PGD) has been recently developed to detect genetic diseases [99,100]. The current methods of sorting single cells include taking trophectoderm cells from blastocyst, blastomeres from embryos at a cleavage stage and polar bodies from the oocyte or zygote [101]. The advantages of a DMF chip include simple accompanying instrumentation and prospective automation for sorting single cells from an early embryo. Huang *et al.* [98] demonstrated a EWOD-based microfluidic device for culture of early mammalian embryos *in vitro*, presaging future clinical application. Although a DMF chip applied in efficient genetic diagnosis is still at an initial stage, we believe that this powerful platform will have a major impact on the ART field.

## 6. Conclusions

Recent developments of microfluidic chips for single cell analysis were reviewed; microfluidic techniques provide numerous advantages for biological and biomedical research, including ease for modularity, small sample requirement, potential of automation, and high-throughput. Perspectives on DMF in analysis of a single cell and efficient genetic screening were particularly focused on and described. We expect that these novel single-cell techniques on microfluidic chips will be important in biomedical areas.
